# Insights into Modifiable Risk Factors of Infertility: A Mendelian Randomization Study

**DOI:** 10.3390/nu14194042

**Published:** 2022-09-28

**Authors:** Wentao Xu, Yueyuan You, Tianqi Yu, Jing Li

**Affiliations:** Research Center of Clinical Epidemiology and Evidence-Based Medicine, West China Hospital, Sichuan University, Chengdu 610041, China

**Keywords:** modifiable risk factors, infertility, Mendelian randomization, causal inference

## Abstract

Objective: Observational studies have linked lifestyle, diet, obesity, and biochemical measures with infertility. Whether this association is causal is unclear. We sought to identify the causal relationship between modifiable risk factors with infertility. Methods: Using single-nucleotide polymorphisms (SNPs) as a genetic instrument variable, we carried out a two-sample Mendelian randomization (MR) analysis to estimate the causal effects for 22 modifiable risk factors on female infertility (6481 cases; 75,450 participants) and male infertility (680 cases; 73,479 participants). Results: The results of the study showed that BMI (OR: 1.24, 95% CI (1.09, 1.40)), body fat percentage (OR: 1.73, 95% CI (1.13, 2.64)), and alcohol consumption (OR: 6.57,95% CI (1.2, 36.14)) are associated with a higher risk of male infertility, and total fatty acids (OR: 1.16, 95% CI (1.03, 1.30), omega-6 fatty acids (OR: 1.14, 95% CI (1.00, 1.27)), and monounsaturated fatty acids (OR: 1.14, 95% CI (1.03, 1.28) are associated with a higher risk of infertility in women. We observed that higher education (OR: 0.77, 95% CI (0.64, 0.92)) was a protective factor for female infertility. Conclusions: BMI, body fat percentage, and alcohol consumption are risk factors for male infertility; total fatty acids, omega-6 fatty acids, and monounsaturated fatty acids are risk factors for female infertility, and education is a protective factor for female infertility.

## 1. Introduction

With the accelerated modernization of society, population problems have become a serious challenge worldwide. Studies by The World Health Organization indicate that the current prevalence of infertility is about 9% [1]. Some scholars believe that the prevalence of infertility is an “iceberg phenomenon” and that most couples suffering from infertility are still undiagnosed [2]. Infertility not only causes greater psychological and social pressure on the couple, but also has an impact on the stability of society [3]. To reduce the social costs of infertility, as well as the public health burden, it is particularly important to identify preventable causes and, in particular, modifiable risk factors [4].

Evidence from observational studies suggests that obesity [5,6,7], smoking [8], alcohol consumption [9], physical activity [10], and dietary habits [11] are associated with infertility. However, due to reverse causality and the presence of confounding factors, conclusions from previous observational studies could be biased. Evidence from animal studies is also unreliable, as exposure measures for risk factors such as smoking, coffee intake, and alcohol consumption are highly different between humans and animals. Therefore, we hope to identify a causal relationship between modifiable risk factors and infertility. Randomized controlled clinical trials (RCTs) are the gold standard for determining causality. However, RCTs are difficult to conduct and often cannot be performed in the vicinity of etiological studies because of medical ethical considerations. Therefore, we used Mendelian randomization (MR) analysis to avoid the limitations of former studies.

MR is an emerging method for epidemiological studies, which uses genetic variants (in this case, single-nucleotide polymorphisms (SNPs)) as instrumental variables (IVs) for causal inference [12]. Since gamete generation follows the Mendelian rule of inheritance, which means that “parental alleles are randomly assigned to offspring”, genetic variants are not influenced by traditional confounding factors, such as environmental exposure, socioeconomic status, and behavioral factors; moreover, genetic variants are inherited from parents and remain unchanged after birth, and their association with outcomes is temporal, so MR can overcome the drawbacks of traditional observational epidemiological studies: unknown confounding factors and reverse causality. With the broad application of genome-wide association studies (GWASs) and GWAS meta-analysis, it is possible to apply MR for causal inference. In this study, we examined the causal relationship between modifiable risk factors and infertility using the two-sample Mendelian randomization design.

## 2. Methods

To increase the sample size and hence statistical efficiency, we performed 2-sample MR using summary-level data from published GWASs.

### 2.1. Data Source

#### 2.1.1. Outcome Data

Summary data on infertility were from FinnGen Consortium R6 release [13]. Male infertility included 835 cases and 85,722 controls; female infertility included 6481 cases and 68,969 controls. Table 1 gives detailed information about the outcome. Infertility diagnostic criteria for diseases are based on ICD8, ICD9, and ICD10.

#### 2.1.2. Exposure Data

We divided modifiable risk factors into 3 categories, obesity-related, lifestyle and dietary factors, and biochemical measures. We conducted a literature review of published studies on infertility (up to 31 May 2022) to identify risk factors for infertility. To be more specific, 3 obesity-related traits (BMI, body fat percentage, waist-to-hip ratio, waist-to-hip ratio adjusted for BMI) [5,6,14], 16 lifestyle and dietary factors (education [15], smoking [8], drinking [16,17], coffee intake [18,19], sleep duration [20,21], insomnia [22], physical activity [5,23], sedentary behavior [24], serum 25-Hydroxyvitamin D levels [25], zinc [26], omega-6 fatty acids, omega-3 fatty acids [27,28], total fatty acids, saturated fatty acids, polyunsaturated fatty acids, monounsaturated fatty acids [29,30]), and 2 biochemical measures (HDL, LDL) were considered as underlying risk factors and were included in a two-sample MR analysis as an exploratory analysis. Table 2 provides details on modifiable risk factors.

#### 2.1.3. IV Selection

We used Single-nucleotide polymorphisms (SNPs) as instrumental variables derived from large GWASs (Table 2). To ensure that the included SNPs were valid SNPs, we set a series of inclusion criteria. We selected SNPs with genome-wide significance (*p* ≤ 5 × 10^−8^) and that had an acceptable probability of mutation (minor allele frequency (MAF) ≥ 3%) without reported loci overlap or linkage disequilibrium (LD) (R^2^ < 0.001). We harmonized all SNPs to ensure that effect estimates corresponded to the same allele. To avoid bias due to weak IVs, we used the F statistic to measure the strength of the IVs. A weak IV was defined as an F statistic less than 10, and all weak instrumental variables were excluded [31]. In addition, palindromic SNPs that would bring ambiguity to the identity of the effect allele in the exposure GWASs were removed. After a series of rigorous screening, the remaining SNPs were considered as eligible IVs.

### 2.2. Statistical Analysis

MR has three core assumptions: (1) the instrument variable (SNPs) is associated with the risk factor; (2) the IV is independent of confounding factors between exposure and the outcome; (3) the IV has no direct effect on the outcome, but only affects the outcome through exposure.

We used inverse variance weighting (IVW), the weighted median (WM), MR-Egger, the weighted model, and the simple model to estimate the causal relationship between exposure (modifiable risk factors) and outcome (infertility).

IVW combines the Wald ratio estimates of each individual SNP into one causal estimate for each risk factor, where the random effects model is used if there heterogeneity exists [32]. Since the selected SNPs might be invalid IVs, the IVW estimates may be biased. We thus further employed four additional analytical models to increase the robustness of the results. First, we used a WM approach, which requires that more than 50% of the weights in the meta-analysis come from valid SNPs [33]. Secondly, MR-Egger was used to detect potential pleiotropy and to correct for the resulting introduced bias [34]. Third, we performed the weighted mode-based estimation method, which requires a smaller sample size and guarantees less bias and lower type-I error rates than other methods. Finally, the simple model-based approach groups SNPs with similar effects into groups based on whether the causal effects are estimated to be similar or not [35].

We used the Cochran Q to test for heterogeneity in these analyses, and we also examined pleiotropy by MR-Egger regression of intercept values and used PhenoScanner (http://www.phenoscanner.medschl.cam.ac.uk/ (accessed on 5 July 2022)) to detect links between genes and other diseases, which was used to exclude gene pleiotropy [13,36].

The results are reported as the ORs and their 95% confidence intervals. We also used two-sided *p* values, and statistical significance existed when *p* < 0.05. All analyses were conducted using the R statistical software version 4.0.2 with the R package “TwoSampleMR”.

## 3. Result

The number of SNPs included per exposure is summarized in Table 2, for results of all analytical methods, please see Supplementary Materials.

### 3.1. Obesity-Related Traits

**Figure 1:**
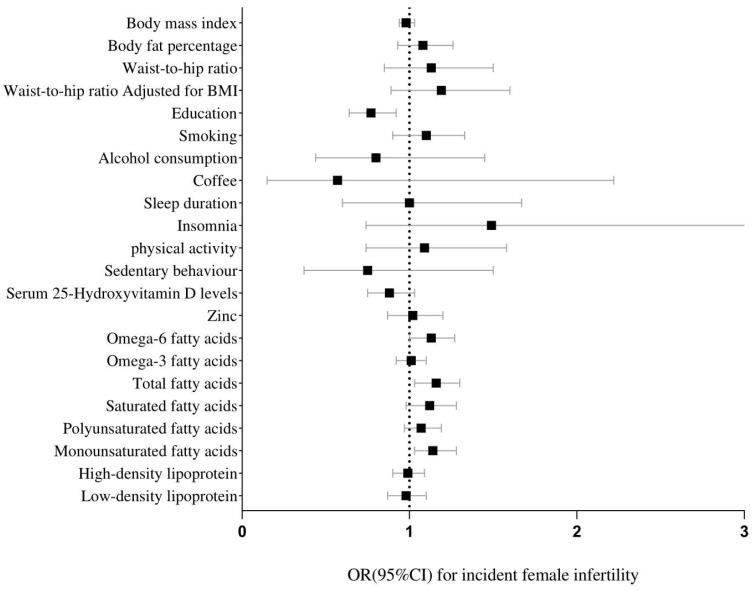
Forest plot of MR analysis between modifiable risk factors and female infertility.

The genetically predicted higher BMI (OR: 1.24, 95%CI (1.09, 1.40)) and body fat (OR: 1.73, 95%CI (1.13, 2.64)), showed a suggestive association with a higher incidence of male infertility risk, but not with female infertility, and MR-Egger showed no pleiotropy (Table 2). In addition, the genetically predicted waist-to-hip ratio and waist-to-hip ratio adjusted for BMI were not associated with female infertility nor male infertility (Table 3 and Figure 1).

### 3.2. Lifestyle and Dietary Factors

**Figure 2:**
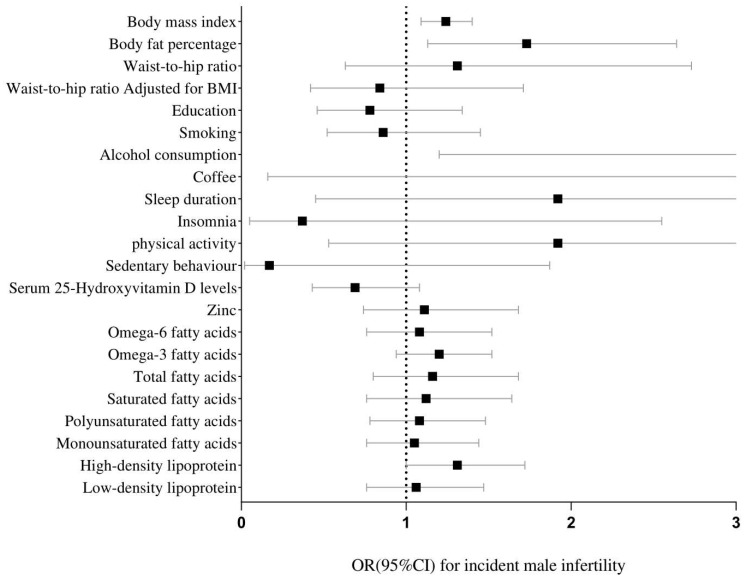
Forest plot of MR analysis between modifiable risk factors and male infertility.

We observed a causal relationship between higher education (OR: 0.004, 95%CI (0.441, 0.321) and lower risk of female infertility, but not with male infertility. The genetically predicted total fatty acids (OR:1.16, 95%CI (1.03, 1.30), omega-6 fatty acids (OR:1.14, 95%CI (1.00, 1.27), and monounsaturated fatty acids (OR:1.14, 95%CI (1.03, 1.28) were related to the risk of female infertility. Meanwhile, we also observed that there was also a causal relationship between alcohol consumption (OR: 6.57, 95%CI (1.2, 36.14) and male infertility (Table 3 and Figure 2). There was no evidence of a potential association between smoking, coffee intake, sleep duration, insomnia, physical activity, sedentary behavior, 25-Hydroxyvitamin D, zinc, and omega-3 fatty acid and the risk of infertility (Table 3 and Figure 1).

### 3.3. Biochemical Measures

No significant results were observed for HDL and LDL (Table 3 and Figure 1). However, IVW showed that there may be a potential causal relationship between HDL (OR: 1.31, 95%CI (1.00, 1.72, *p*: 0.05) and male infertility. Since the *p* value was too close to the threshold, a follow-up study may be needed to verify this conclusion.

The results of all additional analyses can be seen in the Supplementary Material.

## 4. Discussion

To our knowledge, this is the first study to illustrate a causal effect between modifiable risk factors and infertility. We found suggestive associations of the genetically predicted BMI, body fat percentage, and alcohol consumption with male infertility. Furthermore, we found that education, total fatty acids, omega-6 fatty acids, and monounsaturated fatty acids were associated with female infertility.

Our findings are consistent with previous studies. Previous studies have suggested that obesity is a risk factor for infertility [37]. Obesity is a cumulative systemic disease of the entire body, with many mechanisms interacting together to result in a suboptimal environment for sperm production. Hormonal abnormalities associated with obesity blunt the HPG axis, causing a decrease in the intra-testicular testosterone levels required for spermatogenesis [38,39,40]. Increased scrotal temperature due to body habitus and inactivity can also impair semen parameters [41]. Obesity can cause systemic inflammation and elevated levels of inflammatory mediators and reactive oxygen species and cause sperm DNA fragmentation, all of which can lead to infertility [42,43]. We used four obesity-related indicators, two of which exhibited statistical significance and showed a harmful effect. The mechanism of the causal relationship between obesity and infertility is still unclear, and more research may be needed to confirm the relationship in the future.

To our knowledge, no studies have used MR to reveal a causal relationship between education level and infertility. We found a protective effect of education level on the prevalence of female infertility; this is consistent with previous studies [15]. One possible reason is that people with less education may have unhealthy lifestyles, lower socioeconomic status, and poorer medical conditions [44]. In addition, we provided the following evidence for the first time that circulating total fatty acids, omega-6 fatty acids, and monounsaturated fatty acids are causally associated with an increased incidence of female infertility using the MR approach, which is consistent with the findings of previous traditional observational epidemiological studies. This result may be caused by excessive intake of fat in the daily diet and lead to an increase in free fatty acids. Large amounts of free fatty acids may have toxic effects on reproductive tissues, causing cellular damage and a chronic low-grade inflammatory state, which can cause infertility [45].

In our analysis, alcohol consumption is a risk factor for male infertility. Clinical studies have shown that alcohol consumption may alter testosterone production and sperm production. A meta-analysis that included 29,914 researchers showed an association between alcohol and sperm morphology and sperm motility [46]. A cross-sectional survey conducted by Hansen et al. [47] also showed that alcohol consumption was associated with a reduction in most semen parameters. Condorelli et al. [48] also demonstrated that compared to short-term alcohol consumption, infertile patients who consumed alcohol for a long period of time had significantly poorer semen quality and sperm characteristics. However, the biological mechanisms underlying the association between alcohol consumption and male infertility remain poorly understood.

Our study has the following strengths: using MR to assess disease causation effectively avoids unknown confounding factors, as well as reverse causation; data on risk factors are from the largest, latest GWAS; the data were limited to primarily European ancestry cohorts to reduce confounding due to population stratification. More importantly, through large-scale GWAS summary statistics, we investigated a wide range of infertility risk factors that have not been studied in previous MR studies.

Our study has several limitations. First, the explanatory power of genes for exposure may result in a weak IV bias. However, the F statistic for all SNPs was greater than 10, so the possibility of instrumental bias was greatly reduced. Second, although we used MR-Egger methods to detect gene pleiotropy, it is still difficult to exclude the possibility of pleiotropy in causal effects. Third, the efficacy of some of our analyses is limited, so it may lead to false negative results. Additional studies should be conducted subsequently to determine a more accurate association. Finally, our study population was restricted to European ancestry, a setting that reduces the bias of pleiotropy due to ethnic differences, but also leads to findings that may not hold true for other populations.

In conclusion, our analysis provides suggestive evidence that BMI, body fat percentage, and alcohol consumption are risk factors for male infertility; total fatty acids, omega-6 fatty acids, and monounsaturated fatty acids are risk factors for female infertility, and education is a protective factor for female infertility. Our results emphasize the importance of interventions for the primary prevention and management of infertility. Further studies are still needed in the future to draw more accurate conclusions

## Figures and Tables

**Table 1:** Summary of infertility.

Variable	GWAS ID	Population	Number of SNPs	No. Cases	No. Controls	Year	Consortium
Female infertility	finn-b-N14_FEMALEINFERT	European	16,377,038	6481	68,969	2021	FinnGen
Male infertility	finn-b-N14_MALEINFERT	European	16,377,329	680	72,799	2021	FinnGen

**Table 2:** Summary of modifiable risk factors.

Exposure	Unit	Consortium or Study	Sex	Sample Size	Population	No. SNPs	*F* Value	Author and Year
Body mass index	SD (kg/m^2^)	Within family GWAS consortium	Males and females	99,998	European	35	2387	Howe LJe 2022
Body fat	SD (%)	UKB	Males and females	454,850	European	499	45,330	Ben Elsworth 2018
Waist-to-hip ratio	SD	GIANT	Males and females	124,591	European	22	1256	Shungin D 2016
Waist-to-hip ratio adjusted for BMI	SD	GIANT	Males and females	210,082	European	36	2046	Shungin D 2016
Education	years	SSGAC	Males and females	766,345	European	298	15,291	Lee-2018
Smoking	SD	GSCAN	Males and females	607,291	European	84	3850	Liu M 2019
Alcohol consumption	SD	GSCAN	Males and females	335,394	European	50	6489	Liu M 2019
Coffee	SD	MRC-IEU	Males and females	64,949	European	3	127	Ben Elsworth 2018
Sleep duration	SD	UKB Neale Lab	Males and females	334,410	European	42	1649	N
Insomnia	SD	MRC-IEU	Males and females	462,341	European	39	1887	Ben Elsworth 2018
Physical activity	SD	MRC-IEU	Males and females	440,266	European	16	575	Ben Elsworth 2019
Sedentary behavior	SD	UKB	Males and females	91,105	European	4	187	Aiden Doherty 2018
Serum 25-Hydroxyvitamin D levels	SD (nmol/L)	UKB	Males and females	496,946	European	113	23,192	Joana A Revez 2020
Zinc	SD (µmol/L)	1 study	Males and females	2603	European	2	122	Evans David M 2013
Omega-6 fatty acids	SD	1 study	Males and females	114,999	European	52	7014	Borges CM 2020
Omega-3 fatty acids	SD	1 study	Males and females	114,999	European	47	12,039	Borges CM 2020
Total fatty acids	SD	1 study	Males and females	13,505	European	12	627	Kettunen 2016
Saturated fatty acids	SD	1 study	Males and females	114,999	European	49	6315	Borges CM 2020
Polyunsaturated fatty acids	SD	1 study	Males and females	114,999	European	56	8182	Borges CM 2020
Monounsaturated fatty acids	SD	1 study	Males and females	114,999	European	57	8102	Borges CM 2020
High-density lipoprotein	SD (mg/dL)	UKB	Males and females	403,943	European	310	47,586	Richardson, Tom 2020
Low-density lipoprotein	SD (mg/dL)	UKB	Males and females	403,944	European	148	27,197	Richardson, Tom 2021

GIANT: Genetic Investigation of Anthropometric Traits; MRC-IEU: The MRC Integrative Epidemiology Unit at the University of Bristol (IEU); SSGAC: Social Science Genetic Association Consortium; GSCAN: GWAS and Sequencing Consortium of Alcohol and Nicotine use; UKB: UK Biobank.

**Table 3:** Results of MR analysis between modifiable risk factors and infertility.

Exposure	Female Infertility					Male Infertility				
	OR	95%CI	*p*	*p_pleiotropy_*	*p*_heterogeneity_	OR	95%CI	*p*	*p_pleiotropy_*	*p_heterogeneity_*
Body mass index	0.98	(0.94, 1.03)	0.420	0.070	0.834	1.24	(1.09, 1.40)	0.001	0.857	0.375
Body fat percentage	1.08	(0.93, 1.26)	0.286	0.812	0.150	1.73	(1.13, 2.64)	0.011	0.502	0.261
Waist-to-hip ratio	1.13	(0.85, 1.50)	0.400	0.965	0.192	1.31	(0.63, 2.73)	0.475	0.453	0.458
Waist-to-hip ratio adjusted for BMI	1.19	(0.89, 1.60)	0.247	0.294	0.031	0.84	(0.42, 1.71)	0.639	0.370	0.601
Education	0.77	(0.64, 0.92)	0.004	0.441	0.321	0.78	(0.46, 1.34)	0.372	0.667	0.404
Smoking	1.10	(0.90, 1.33)	0.360	0.450	0.060	0.86	(0.52, 1.45)	0.580	0.164	0.409
Alcohol consumption	0.80	(0.44, 1.45)	0.463	0.699	0.327	6.57	(1.20, 36.14)	0.030	0.834	0.434
Coffee	0.57	(0.15, 2.22)	0.418	0.390	0.347	8.08	(0.16, 398.68)	0.294	0.605	0.717
Sleep duration	1.00	(0.60, 1.67)	0.991	0.203	0.344	1.92	(0.45, 8.23)	0.377	0.812	0.649
Insomnia	1.49	(0.74, 3.01)	0.260	0.466	0.034	0.37	(0.05, 2.55)	0.310	0.944	0.099
Physical activity	1.09	(0.74, 1.58)	0.460	0.042	0.113	1.92	(0.53, 6.93)	0.322	0.136	0.014
Sedentary behavior	0.75	(0.37, 1.50)	0.417	0.851	0.817	0.17	(0.02, 1.87)	0.147	0.384	0.247
Serum 25-Hydroxyvitamin D levels	0.88	(0.75, 1.03)	0.122	0.439	0.429	0.69	(0.43, 1.08)	0.105	0.541	0.604
Zinc	1.02	(0.87, 1.20)	0.795	\	0.204	1.11	(0.74, 1.68)	0.615	\	0.269
Omega-6 fatty acids	1.13	(1.00, 1.27)	0.046	0.722	0.505	1.08	(0.76, 1.52)	0.682	0.432	0.609
Omega-3 fatty acids	1.01	(0.92, 1.10)	0.888	0.244	0.148	1.20	(0.94, 1.52)	0.140	0.533	0.590
Total fatty acids	1.16	(1.03, 1.30)	0.015	0.763	0.885	1.16	(0.80, 1.68)	0.443	0.644	0.298
Saturated fatty acids	1.12	(0.98, 1.28)	0.090	0.562	0.288	1.12	(0.76, 1.64)	1.646	0.456	0.289
Polyunsaturated fatty acids	1.07	(0.97, 1.19)	0.185	0.544	0.492	1.08	(0.78, 1.48)	0.651	0.130	0.374
Monounsaturated fatty acids	1.14	(1.03, 1.28)	0.015	0.196	0.688	1.05	(0.76, 1.44)	0.785	0.669	0.783
High-density lipoprotein	0.99	(0.90, 1.09)	0.831	0.225	0.344	1.31	(1.00, 1.72)	0.053	0.944	0.251
Low-density lipoprotein	0.98	(0.87, 1.10)	0.735	0.279	0.141	1.06	(0.76, 1.47)	0.728	0.662	0.255

## Data Availability

The data involved in this study can be downloaded from http://gwas.mrcieu.ac.uk.

## Supplementary Materials

The following supporting information can be downloaded at: https://www.mdpi.com/article/10.3390/nu14194042/s1, Table S1: Detailed results of the above 5 statistical methods.
