# Supplementary figures and images for: Insights into Modifiable Risk Factors of Infertility: A Mendelian Randomization Study
Source: Nutrients. 2022 Sep 28;14(19):4042. doi: 10.3390/nu14194042 (PMC9572512; doi:10.3390/nu14194042)

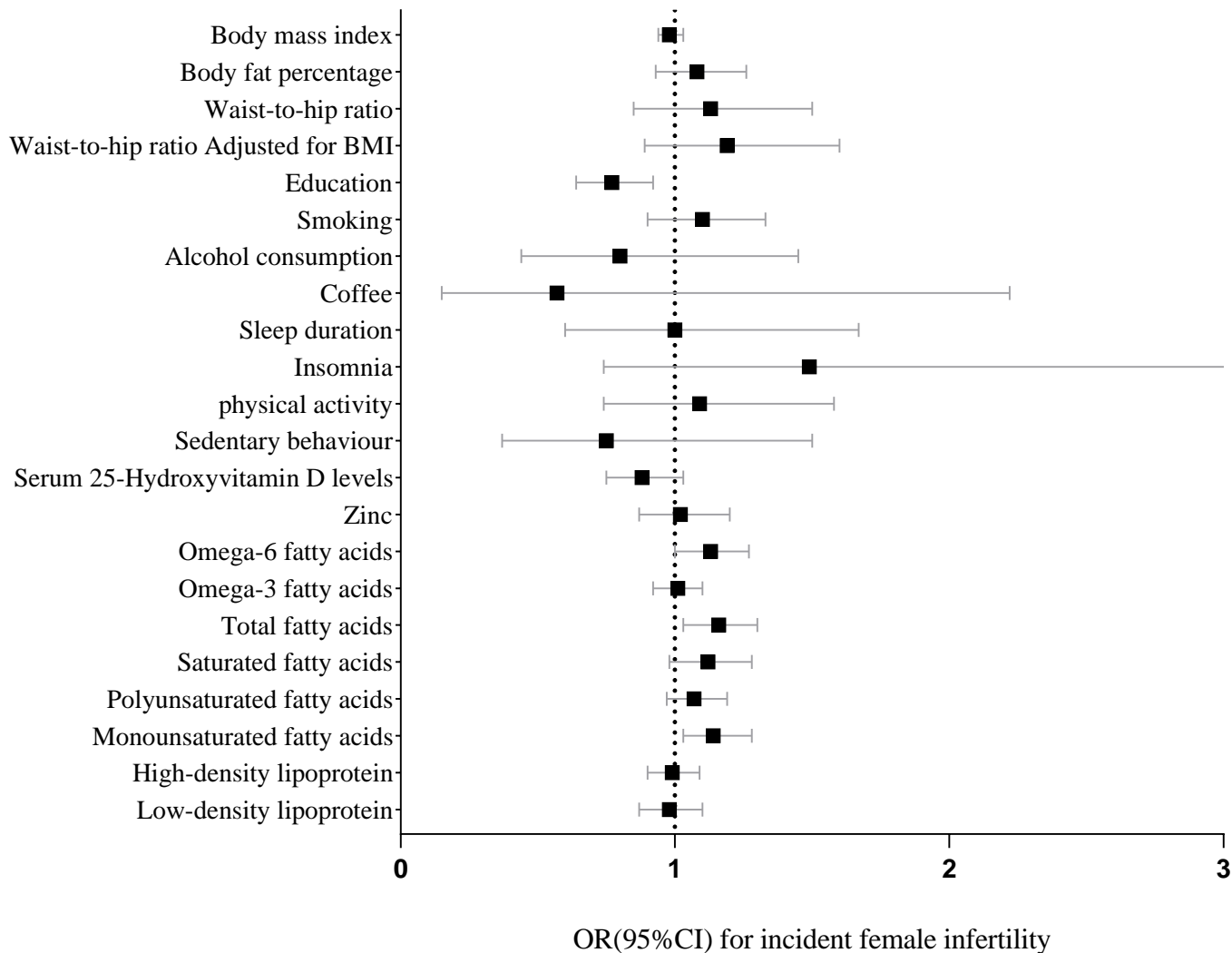

Supplement: Supplementary file 1 [file nutrients-14-04042-s001.zip › Odds ratios famale.pdf]

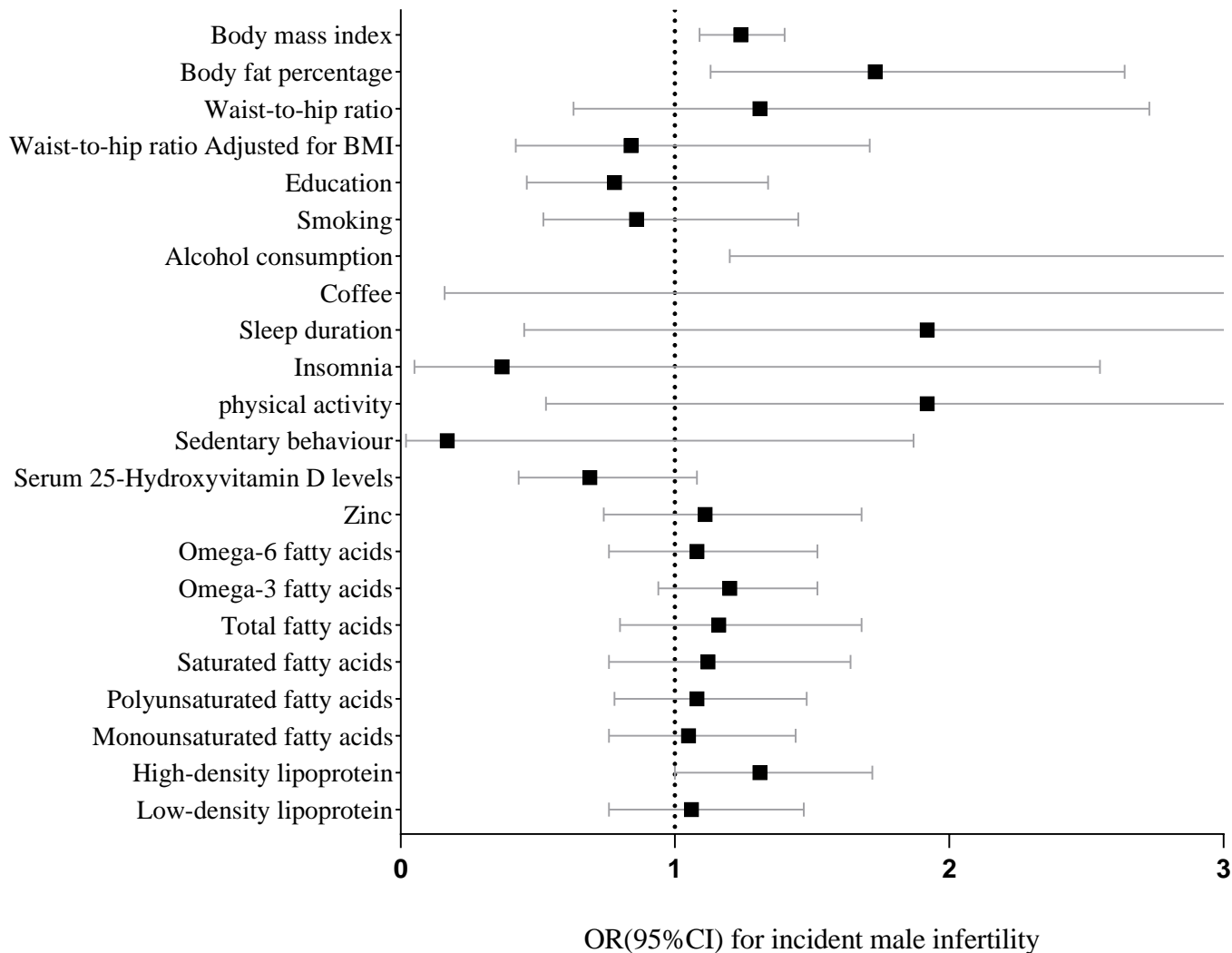

Supplement: Supplementary file 1 [file nutrients-14-04042-s001.zip › Odds ratios male.pdf]
